# ﻿Description of a new montane freshwater crab (Arthropoda, Malacostraca, Decapoda, Potamonautidae) from the Eastern Cape, South Africa

**DOI:** 10.3897/zookeys.1160.100844

**Published:** 2023-05-04

**Authors:** Nasreen Peer, Gavin Gouws, Lazola Maliwa, Nigel Barker, Paul Juby, Renzo Perissinotto

**Affiliations:** 1 Department of Botany and Zoology, Stellenbosch University, Merriman Avenue, Stellenbosch, 7600, South Africa Stellenbosch University Stellenbosch South Africa; 2 National Research Foundation – South African Institute for Aquatic Biodiversity, Private Bag 1015, Grahamstown, 6140, South Africa National Research Foundation – South African Institute for Aquatic Biodiversity Grahamstown South Africa; 3 Albany Museum, 40 Somerset Street, Grahamstown, 6139, South Africa Albany Museum Grahamstown South Africa; 4 Department of Plant and Soil Sciences, University of Pretoria, Private Bag X20, Hatfield, 0028, Pretoria, South Africa University of Pretoria Pretoria South Africa; 5 Institute for Coastal & Marine Research, Nelson Mandela University, Gqeberha, 6031, South Africa Nelson Mandela University Gqeberha South Africa

**Keywords:** Afrotropical Region, Brachyura, high altitude streams, molecular analyses, morphology, taxonomy

## Abstract

A new species of freshwater crab, *Potamonautesamathole***sp. nov.**, is described from the Winterberg-Amathole mountain range in the Eastern Cape Province, South Africa. Morphologically, *P.amathole* Peer & Gouws, **sp. nov.** most closely resembles *P.tuerkayi* but can be distinguished by key morphological characters including the variation in the shape of the subterminal segment of gonopod 2 between both species. Genetically, *P.amathole* Peer & Gouws, **sp. nov.** is placed within the clade of small-bodied, mountain-dwelling crabs including *P.parvispina*, *P.parvicorpus*, *P.brincki*, *P.tuerkayi*, *P.baziya*, and *P.depressus*. The new species is found in slow-moving mountain streams and pools at high altitudes. The continued discovery and description of new freshwater crab species reinforces the need for ongoing research, especially in under-sampled regions.

## ﻿Introduction

In South Africa, the genera *Potamonautes* and *Maritimonautes* represent the freshwater crabs with a total of 26 described species since the last published descriptions (Daniels et al. 2022). Although the freshwater crabs in South Africa are a fairly well-studied group, morphological and molecular analyses continue to reveal new undescribed species, many of which are cryptic and have thus been mistaken for previously described species (Daniels et al. 1998, 2022; Phiri and Daniels 2014, 2016; Peer et al. 2017).

Recently, several new species have been described from natural forest habitats (Peer et al. 2015; Daniels 2017; Daniels et al. 2019, 2021), which only comprise a small percentage of South Africa’s total land cover (0.4%) but boast the highest biodiversity per hectare (Geldenhuys and MacDevette 1989). Although mostly fragmented, the Knysna and Amathole Forest complexes are the two largest remaining patches of natural forest in South Africa, with the latter situated in the Amathole Mountain Range along the Great Escarpment (Mucina and Rutherford 2006). The Great Escarpment, as outlined by Clark et al. (2011a), refers to a semi-continuous mountain range that transcends many southern African borders, experiencing a range of climatic conditions.

Aside from high forest biodiversity, the Amathole Mountain Range, forming part of the escarpment between the Sneeuberg range in the west and the Drakensberg range in the east, is considered to display great diversity across habitat types, as well as high levels of endemism in terms of flora (Clark et al. 2011a). Despite this, habitats in the region are still poorly sampled in terms of fauna (Kok et al. 2012; Taylor et al. 2020).

In this paper, we describe *P.amathole* sp. nov. from the Hogsback and Katberg Forests in the Amathole Mountains of the Eastern Cape. NP and GG wrote the taxonomic part of this study, including the description of the new species, while the contribution of the other authors dealt with genetic analyses, natural history, and ecological observations.

## ﻿Materials and methods

### ﻿Crab collection

**Figure 1.**
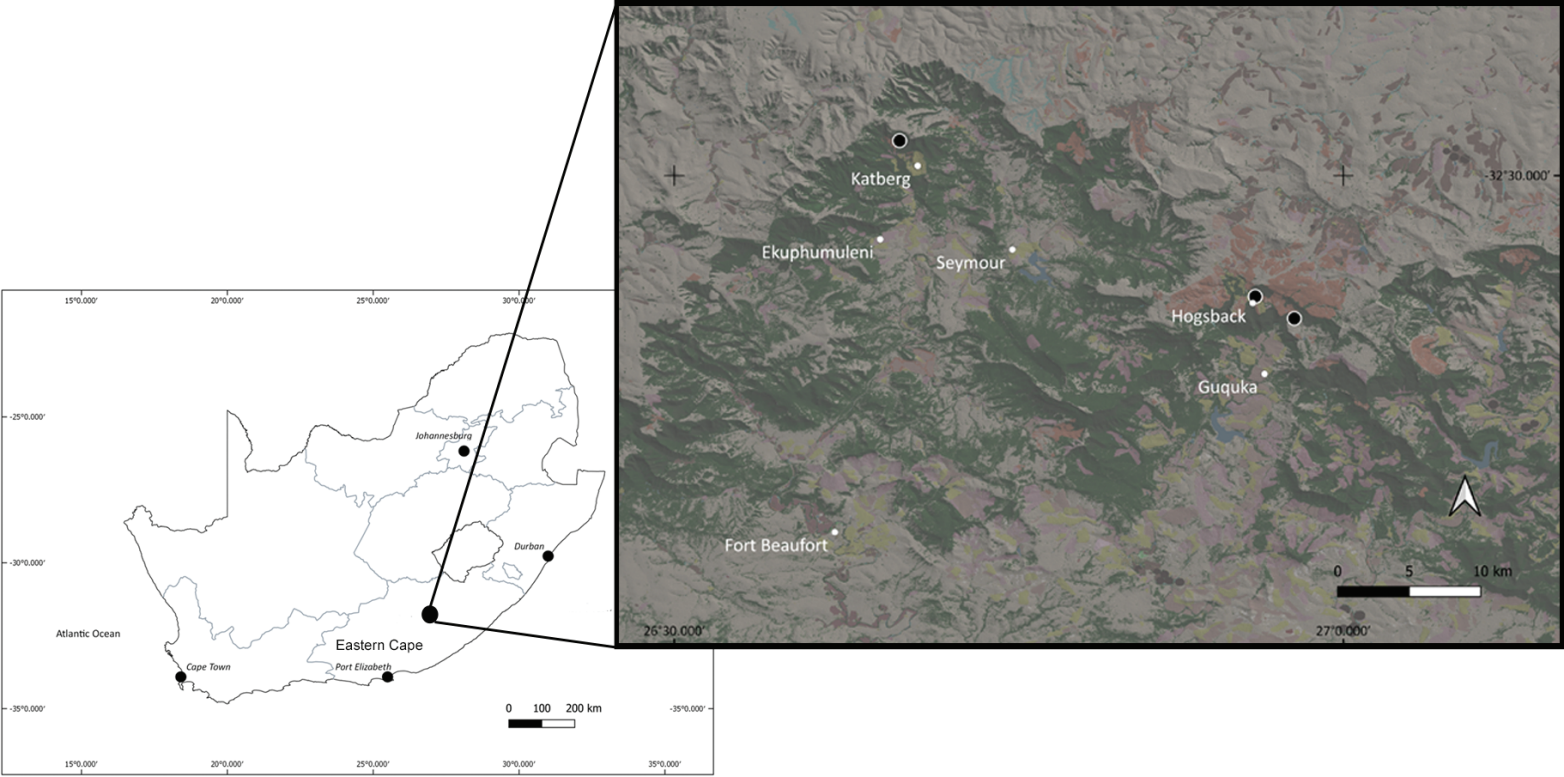
Map showing the location of the Amathole Mountain Range sampling sites (black markers) in relation to nearby towns and villages (white points). South Africa National Land Cover (SANLC) 2018 map layer shows land cover to be predominantly indigenous forest and plantations with residential area nearby.

Crabs were collected from three localities (Table 1, Fig. 1) located in the Winterberg-Amathole mountain range.

**Table 1.** Localities of the three sample sites.

Site name	Coordinates
Katberg State Forest	32°28.43'S, 26°40.10'E
Hogsback Arboretum	32°35.42'S, 26°56.05'E
Hogsback Waterfall	32°36.40'S, 26°57.80'E

Crabs were collected by hand or net and preserved in 70% ethanol.

### ﻿Genetic analyses

The genetic placement and relationships of the proposed new species were examined using data generated in two sequential studies.

For the first study, total genomic DNA was extracted from 0.5–1 mg of pereiopod muscle tissue from specimens collected from Hogsback and Katberg during 2013. Tissue was rinsed in sterile water and DNA was extracted using an Invisorb Spin Tissue Mini Kit (Invitek Molecular, Berlin), following the manufacturer’s protocol.

A fragment of the large ribosomal subunit 16S mitochondrial marker was amplified by Polymerase Chain Reaction, using the primers of Cunningham et al. (1992: 16Sar and 16Sbr). PCRs were completed in 50 µL volumes, comprising 1 X PCR reaction buffer, 5 µL template DNA, 2 mM MgCl_2_, 0.2 mM deoxynucleotide triphosphate (dNTP), 0.2 µM of each primer, and 0.5 U *Taq* polymerase. The thermocycling profile included an initial denaturing at 95 °C for 5 min, 30 cycles of 95 °C for 45 s, 45 °C for 45 s and 72 °C for 1.5 min, followed by a final extension of 72 °C for 5 min. Amplification was confirmed by gel electrophoresis in a 0.5% agarose gel stained with SYBR Green (ThermoFisher Scientific, Waltham, Massachusetts) and viewed with a UV-transilluminator. PCR products were purified with an Invisorb PCRapace Quick purification kit (Invitek Molecular). Purified products were cycle-sequenced in both forward and reverse directions using the ABI Big Dye Sequencing kit v. 3.1. (Applied Biosystems, Austin, Texas). Cycle-sequencing products were precipitated using a NaAc-ethanol procedure (Sambrook and Russell 2001) and analysed on an ABI 3100 Genetic Analyser at Rhodes University, South Africa. Consensus sequences were created from the forward and reverse sequences of each sample, correcting base ambiguities, using SEQUENCHER v. 4.5 (GeneCodes Corporation).

In the second study, DNA was extracted from specimens collected from Hogsback during 2018, using a PureLink Miniprep kit (Invitrogen, Carlsbad, California). A fragment of the protein-coding mtDNA cytochrome *c* oxidase subunit I (COI) gene was amplified and sequenced using the approach described by Gouws et al. (2015). Sequences were checked and edited as described therein.

### ﻿Data analyses

Data sets for each of the 16S and COI fragments included data generated in the present study and published data for all described southern African *Potamonautes* species, as compiled previously (Gouws et al. 2015). GenBank accession numbers and sources for the published data are provided in Suppl. material 1: table S1. *Potamonautesdanielsi*, described by Peer et al. (2017), was represented by an individual (NPP2) from Network E within a wider lineage of *P.sidneyi* in the study by Gouws et al. (2015); this individual was sampled from the type locality (Mtamvuna River) of *P.danielsi*. Similarly, the *P.brincki* sequences included by Gouws et al. (2015) were ascribed to *P.tuerkayi* in the present study. The latter species was described as distinct from *P.brincki* by Wood and Daniels (2016) with the type locality being Fernkloof, from where the aforementioned *P.brincki* specimens were sampled for the study by Daniels et al. (2002). An alternative COI sequence (Wood and Daniels 2016: GenBank accession number KU561507) was included for *P.brincki*; unfortunately, no other 16S sequence was available. The *P.lividus* specimen from Eswatini (formerly Swaziland) included in the study by Gouws et al. (2015) has subsequently been assigned to *P.valles* (Daniels et al. 2022). The previously-compiled data were also supplemented with data for other South African species described subsequent to the Gouws et al. (2015) study, including *P.baziya* (Daniels et al. 2021: GenBank accession number 16S – OK482902, COI – OK489798), *P.mariepskoppie* (Daniels et al. 2021: 16S – OK482901, COI – OK489797), *P.ngoyensis* (Daniels et al. 2019: 16S – MK607207, COI – MK607221), *P.ntendekaensis* (Daniels et al. 2019: 16S – MK607196, COI – MK607210) and *P.mhlophe* (Daniels 2017: 16S – MF693159, COI – MF693167). In a recent systematic revision, Cumberlidge and Daniels (2022) reassigned several of the *Potamonautes* species included by Gouws et al. (2015) to the genera *Arcopotamonautes* (*A.bellarussus*, *A.lirrangensis*, *A.platynotes* and *A.raybouldi*), *Maritimonautes (M.calcaratus*, *M.choloensis*, *M.namuliensis* and *M.obesus*) and *Rotundopotamonautes* (*R.ohdneri* and *R.subukia*). The representatives of *Maritimonautes* were used as outgroups in the present study, due to their basal placement relative to *Potamonautes* (Cumberlidge and Daniels 2022). Representatives of other genera were excluded from these analyses.

ClustalX2 (Larkin et al. 2007) was used to align the COI partition, while the 16S data were aligned using MAFTT 6.956 (Katoh and Toh 2008) with an iterative refinement strategy (L-INS-i) (Katoh et al. 2005). As corresponding 16S and COI sequences were not successfully obtained for every individual included in the present study, the 16S and COI data sets were analysed separately. Phylogenetic relationships were determined using Maximum Likelihood (ML) analyses, as implemented in PAUP*4.0a168 (Swofford 2003). Prior to execution, the optimal models of nucleotide substitution for each of the 16S and COI data sets were identified using jModelTest 2.1.4 (Darriba et al. 2012), with model selection determined using the Akaike (1974) Information Criterion. In the ML analyses, heuristic tree searches were executed, with TBR branch-swapping of a tree obtained through a random addition of taxa, with 100 such replicates employed. Due to computational constraints, nodal support was evaluated through Bayesian Posterior Probabilities (BPPs), with values above 0.95 being regarded as evidence of support. These were generated through Bayesian inferences as described in Gouws et al. (2015) but sampling the posterior distribution every 5 000 generations. Uncorrected sequence divergences among individuals were calculated using PAUP.

### ﻿Morphological measurement and description

Morphological variables were measured using a pair of Vernier callipers. A Canon Powershot G12 digital camera was used to photograph carapaces and appendages, while a Nikon SMZ25 microscope fitted with a Nikon Digital Sight DS-Fi2 camera was used for macro-examination and to take photos of gonopods and mouthparts.

### ﻿Abbreviations for depositories and provinces

**ISAM**Iziko South African Museum, Cape Town, South Africa;

**EC** Eastern Cape;

**WC** Western Cape;

**KZN** KwaZulu–Natal.

Abbreviations for all morphological and morphometric characters (following Gouws et al. 2001):

**AW6** Width of sixth abdominal segment;

**CH** Carapace height;

**CL** Carapace length;

**CLDL** Left cheliped, dactyl length;

**CRDL** Right cheliped, dactyl length;

**CRPL** Right cheliped, propodus length;

**CRPW** Right cheliped, propodus width;

**CWA** Distance between exorbital teeth;

**CWW** Carapace widest width;

**CWP** Carapace posterior width;

**ED** Distance between orbits;

**MCPL** Major cheliped propodus length;

**MCPW** Major cheliped propodus width;

**ML** Merus length;

**MW** Merus width;

**PFCD** Distance between postfrontal crest and anterior margin;

**P2ML** Pereopod 2, merus length;

**P2MW** Pereopod 2, merus width;

**s2/s3** First sternal groove (suture between the second and third sulci);

**s3/s4** Second sternal groove (suture between the third and fourth sulci).

## ﻿Results

### ﻿Genetic analyses

**Figure 2.**
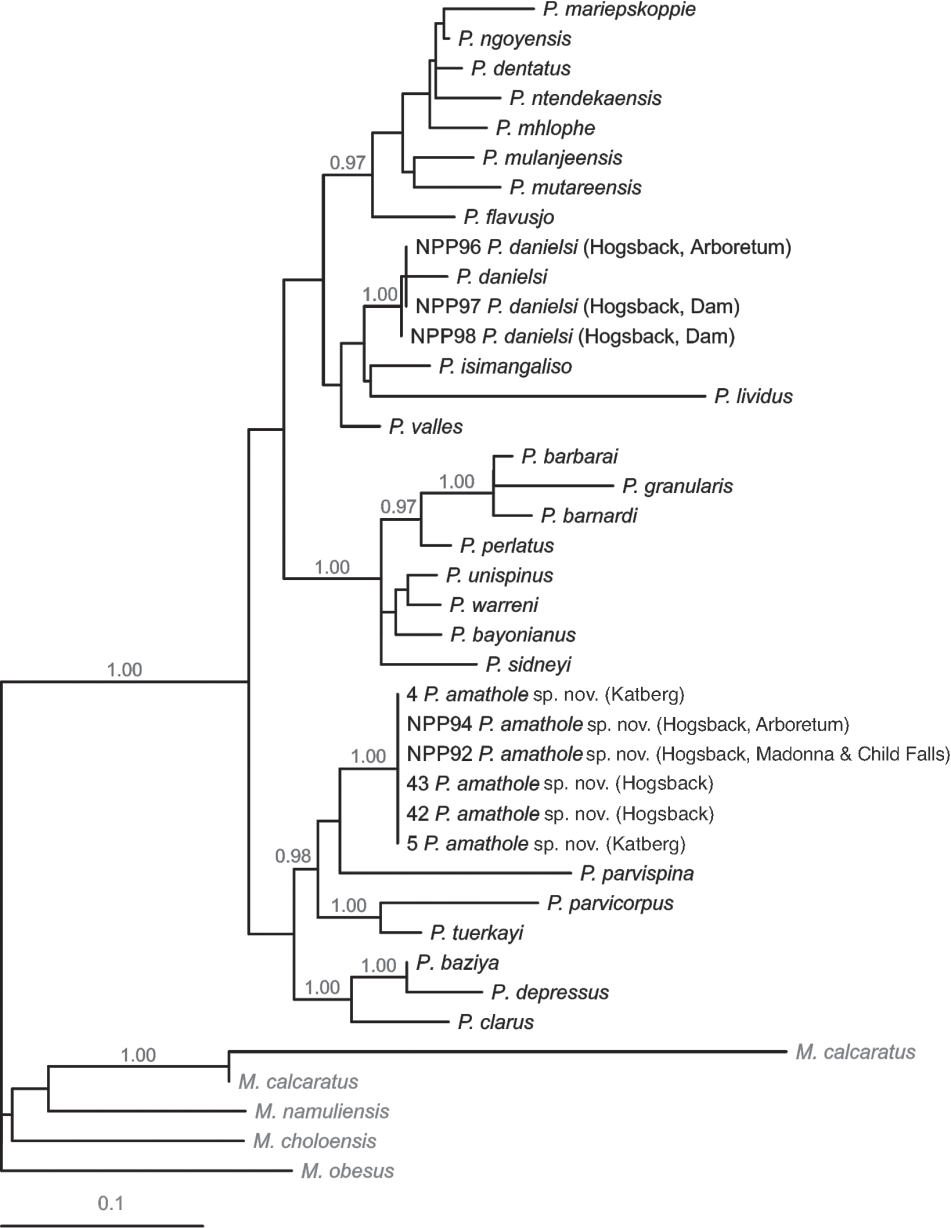
The most likely topology (-lnL = 3754.743) obtained in the Maximum-Likelihood analysis of a 549 nucleotide 16S rRNA alignment, depicting relationships between potamonautid freshwater crabs sampled from Hogsback and Katberg (Eastern Cape, South Africa) and known southern African *Potamonautes* species. Nodal support in the form of Bayesian Posterior Probabilities (BPPs) are indicated above the branches, with only BPPs > 0.95 shown. Species of *Maritimonautes* are included as outgroups.

New sequences generated in the present study were lodged in GenBank (16S: accession numbers OQ559329–OQ559337; COI: OQ558909–OQ558911). The 16S alignment was 549 nucleotides in length. The ML analysis, using the parameters of the optimal model (base frequencies: A = 0.369, C = 0.122, G = 0.163 and T = 0.346; rate matrix: R_A↔C_ = 0.571, R_A↔G_ = 5.756, R_A↔T_ = R_G↔T_ = 1.000, R_C↔G_ = R_C↔T_ = 2.251; α = 0.341 for the gamma distribution of rate variation), produced the topology (-lnL = 3754.743) presented in Fig. 2.

**Figure 3.**
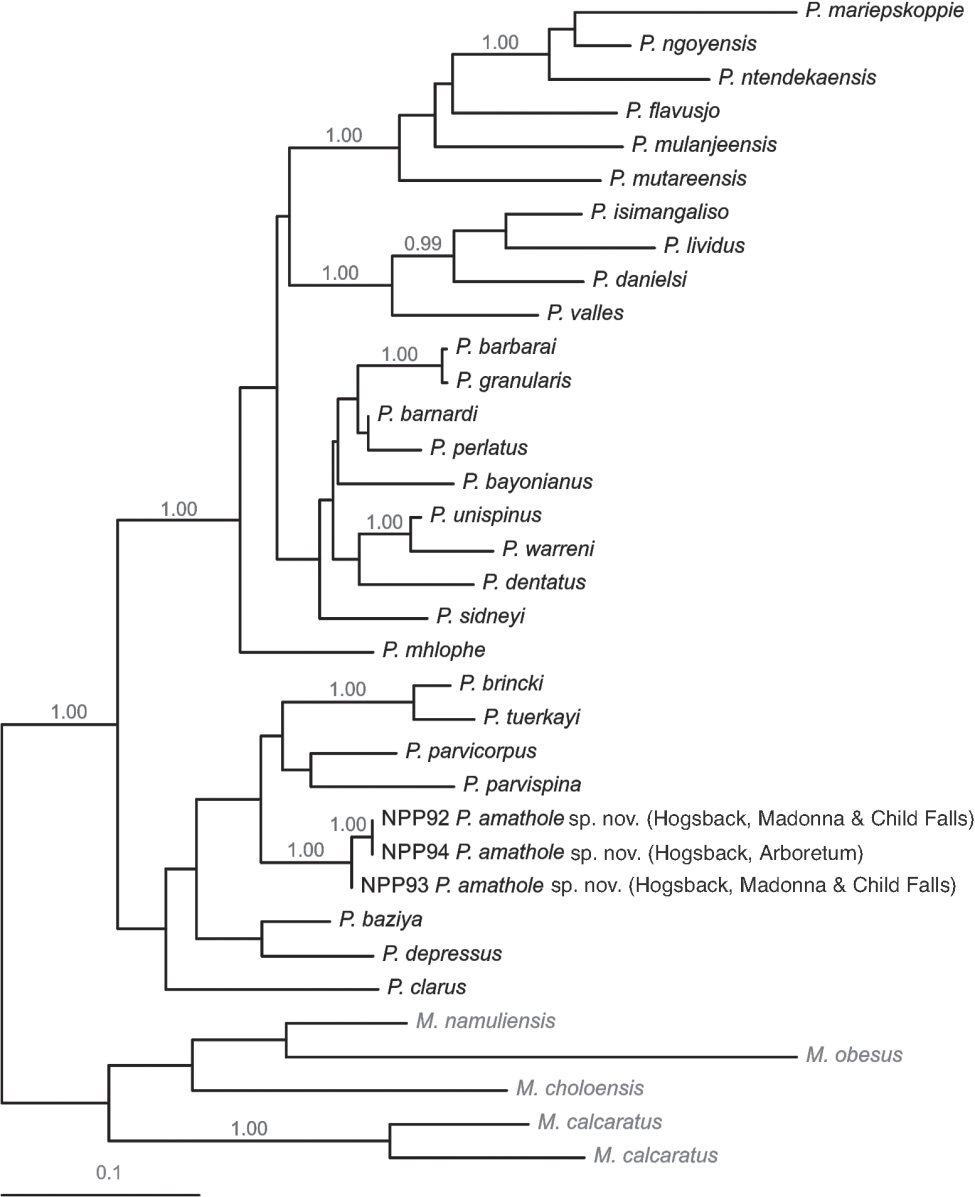
The most likely topology (-lnL = 5093.453) obtained in the Maximum-Likelihood analysis of a 660 nucleotide mtDNA cytochrome *c* oxidase subunit I (COI) alignment, depicting relationships between potamonautid freshwater crabs sampled from Hogsback (Eastern Cape, South Africa) and known southern African *Potamonautes* species. Nodal support in the form of Bayesian Posterior Probabilities (BPPs) are indicated above the branches, with only BPPs > 0.95 shown. Species of *Maritimonautes* are included as outgroups.

The COI alignment was 660 nucleotides in length. The optimal model had base frequencies of A = 0.292, C = 0.184, G = 0.154 and T = 0.371, a rate matrix of R_A↔C_ = 4.203, R_A↔G_ = 10.249, R_A↔T_ = 2.385, R_C↔G_ = R_G↔T_ = 1.000, R_C↔T_ = 31.047, a proportion of invariant sites (I = 0.549) and a gamma distribution of rate variation (α = 1.280). The tree produced by the ML analysis is shown as Fig. 3.

The topologies produced by the analyses of the 16S and COI data were congruent with respect to, and reflected, the major phylogenetic divisions reported previously for the southern African *Potamonautes* radiation (e.g., Daniels 2017; Daniels et al. 2019, 2021; Cumberlidge and Daniels 2022). These include a clade of small-bodied, mountain-dwelling crabs i.e., *Potamonautesbaziya* Daniels, Barnes, Marais & Gouws, 2021, *P.brincki* (Bott, 1960), *P.clarus* Gouws, Stewart & Coke, 2000, *P.depressus* (Krauss, 1843), *P.parvicorpus* Daniels, Stewart & Burmeister, 2001, *P.parvispina* Stewart, 1997 and *P.tuerkayi* Wood & Daniels, 2016; a clade of robust, large-bodied riverine species i.e. *P.barbarai* Phiri & Daniels, 2014, *P.barnardi* Phiri & Daniels, 2014, *P.bayonianus* (de Britto Capello, 1864), *P.dentatus* Stewart, Coke & Cook, 1995, *P.granularis* Daniels, Stewart & Gibbons, 1998, *P.perlatus* (H. Milne Edwards, 1837), *P.sidneyi* Rathbun, 1904, *P.unispinus* Stewart & Cook, 1998 and *P.warreni* Calman, 1918); a clade of species largely inhabiting forests in the Indian Ocean Coastal Belt (IOCB) (*P.danielsi* Peer & Gouws, 2017, *P.isimangaliso* Peer & Gouws, 2015, *P.lividus* Gouws, Stewart & Reavell, 2001 and *P.valles* Daniels, Busschau, Gullacksen, Marais, Gouws & Barnes, 2022); and a clade of mostly burrowing IOCB or tropical highland species with *P.flavusjo* Daniels, Phiri & Bayliss, 2014, *P.mariepskoppie* Daniels, Barnes, Marais & Gouws, 2021, *P.mulanjeensis* Daniels & Bayliss, 2012, *P.mutareensis* Phiri & Daniels, 2013, *P.ngoyensis* Daniels, Busschau & Cumberlidge, 2019 and *P.ntendekaensis* Daniels, Busschau & Cumberlidge, 2019. Discrepancies between the two topologies concerned the placement of *P.dentatus* and *P.mhlophe* Daniels, 2017 (see Figs 2 and 3); the generally accepted phylogenetic placement of these species is within the last of the aforementioned clades (see Daniels 2017; Daniels et al. 2019, 2021; Cumberlidge and Daniels 2022), as in the 16S topology.

Of relevance to the current study, the crabs sampled from Hogsback and Katberg were placed in two separate clades. In the 16S topology, samples from Hogsback and Katberg were placed within the clade of small-bodied, mountain-dwelling species, sister to *P.parvispina*, in a larger well-supported clade (BPP = 0.98) of mountain-dwelling species from the Western Cape (*P.parvicorpus*, *P.parvispina*, and *P.tuerkayi*), which was sister to a clade containing those (*P.baziya*, *P.clarus*, and *P.depressus*) from the Drakensberg in the Eastern Cape and KwaZulu-Natal. Other samples from Hogsback, including one collected sympatrically with the aforementioned at the Hogsback Arboretum, were allied to *P.danielsi* within the clade of IOCB forest-dwelling species with high support (BPP 1.00). In the COI topology, where only samples from Hogsback were included, these were placed similarly within the clade of mountain-dwelling species. These results suggest the presence of two distinct species among the crabs sampled from Hogsback and Katberg.

Uncorrected sequence divergences among individuals are presented in Suppl. material 1: table S2. The Hogsback specimens within the clade of IOCB species were 1.5% divergent from *P.danielsi*. Given their close affinity and the general trends in terms of sequence divergence, and the strong support (BPP = 1.00) for the clade formed by these specimens and *P.danielsi*, it is considered that these are conspecific. The Hogsback and Katberg specimens within the mountain-dwelling clade were 5.4 to 8.9% divergent from the other species in this clade and were 5.8 to 8.3% divergent from their sister-taxon (*P.parvispina*). In the context of the above divergences among known species, the Hogsback and Katberg specimens in this clade are considered to be a distinct species, described below. The Hogsback specimens were also 7.6–8.0% divergent from those collected in sympatry or near-sympatry but belonging to the IOCB clade.

For the COI data, where the only specimens included from Hogsback belonged to the clade of mountain-dwelling species, uncorrected sequence divergences between these specimens and other species in that clade ranged from 7.4 to 10.9%. With the exception of the comparison between *P.barbarai* and *P.granularis* (0.5%), previously described species were 2.4 to 18.4% divergent. Comparatively, these divergences again support the taxonomic distinctiveness of the Hogsback (and, by extension, Katberg) specimens.

### ﻿Taxonomic description

**﻿Genus *Potamonautes***

#### ﻿ Potamonautes amathole

Taxon classificationAnimaliaDecapodaPotamonautidae

Peer & Gouws sp. nov.

0FDA1948-A61F-5785-B804-4500713FA65B

https://zoobank.org/E47B3AFA-479B-496E-B7E3-F1F13849CEC2

##### Type series.

***Holotype***: male, CL = 25.3 mm (Table 2), cascading stream in the Katberg State Forest (32°28'26.4"S, 26°40'05.9"E, elevation 1070 m), 25 October 2018, L. Maliwa, N. Miranda and N. Peer legit (MB-A094813). Allotype: female, CL = 24.9 mm (Table 2), collection details as per holotype (MB-A094814). ***Paratypes***: (Table 2) collection details same as above, MB-A094815 (8 ♂, 4 ♀). Hogsback Arboretum, MB-A094816 (2 ♀), 32°35'25.2"S, 26°56'02.7"E, elevation 1235 m, 24 October 2018, L. Maliwa, N. Miranda and N. Peer legit. Madonna and Child Waterfall, Hogsback, MB-A094817 (1 ♂, 1 ♀); 32°36'24.4"S, 26°57'48.2"E, elevation 1092 m, 24 October 2018, L. Maliwa, N. Miranda and N. Peer legit.

**Table 2.** Ranges of measurements (mm) for 12 morphometric variables of the *Potamonautesamathole* sp. nov. holotype and paratypes collected from Katberg and Hogsback, as well as *P.brincki*, *P.tuerkayi*, *P.parvispina*, and *P.parvicorpus* from published sources.

Variable	*Potamonautesamathole* sp. nov.			*Potamonautesbrincki* Stewart, 1997		*Potamonautestuerkayi* Wood & Daniels, 2016	*Potamonautesparvispina* Stewart, 1997		*Potamonautesparvicorpus* Daniels, Stewart & Burmeister, 2001	
	Holotype	Males (*n* = 11)	Females (*n* = 8)	Males	Females	Holotype	Males	Females	Males	Females
CL	25.3	19.5–25.3	19.3–25.5	15.5–26.9	9.1–25.3	20	20.8–28.7	22.7–28.4	11.85–24.08	9.13–24.71
CWW	37.3	27.8–37.5	26.8–35.5	20.4–38.2	11.4–35.1	30	28.9–42.0	31.5–41.3	15.9–36.24	11.41–34.99
CWP	12.5	10.0–14.3	10.5–15.0	16.8–28.6	9.8–27.7	10	23.3–32.6	25.6–32.8	12.75–26.38	9.84–27.95
CH	12.8	9.0–12.8	8.5–12.0	4.5–7.7	4.5–13.6	10	10.3–16.4	12.4–15.3	5.68–13.48	4.47–13.12
PFCD	3.8	3.0–3.8	2.5–3.8	2.6–3.9	1.4–3.6	4	3.2–4.6	3.7–4.5	2.06–4.27	1.42–3.82
ED	13.5	9.5–13.8	9.0–12.5	7.7–13.4	4.7–12.4	-	10.7–15.3	11.7–14.5	7.03–13.28	4.73–14.07
CWA	25	18.5–24.5	17.8–23.8	15.1–26.5	9.3–24.6	10	21.7–29.2	22.2–27.6	12.57–24.09	9.27–23.84
AW6	8	6.0–7.8	15.0–21.8	13.1–42.7	6.6–22.7	-	5.6–8.1	13.5–24.7	3.62–7.63	3.10–21.57
MCPL	39	22.0–37.0	15.0–21.5	5.1–17.7	2.4–8.4	32	19.8–42.1	19.2–24.2	9.60–25.08	6.59–19.15
MCPW	16.1	9.0–15.3	5.0–7.3	8.2–17	4.5–13.9	12	8.0–19.4	7.3–9.1	3.34–12.81	2.41–10.84
P2ML	15	11.0–15.5	8.8–12.5	7.8–14.9	3.1–23.2	12	11.2–18.5	12.7–14.8	6.21–14.20	1.92–12.62
P2MW	4	3.0–4.0	3.0–4.3	3.6–5.9	1.9–5.6	3	4.4–5.5	5.2–5.4	2.76–4.77	1.92–4.44

##### Diagnosis.

*Potamonautesamathole* sp. nov. exhibits a smooth carapace, flank and epibranchial region, with a rounded anterolateral margin and a narrow posterior end. Postfrontal crest complete. Dactyl of major cheliped highly arched. Pereopod 4 is longest. Bi-lobed maxillary palp with no flange.

##### Description of holotype.

**Figure 4.**
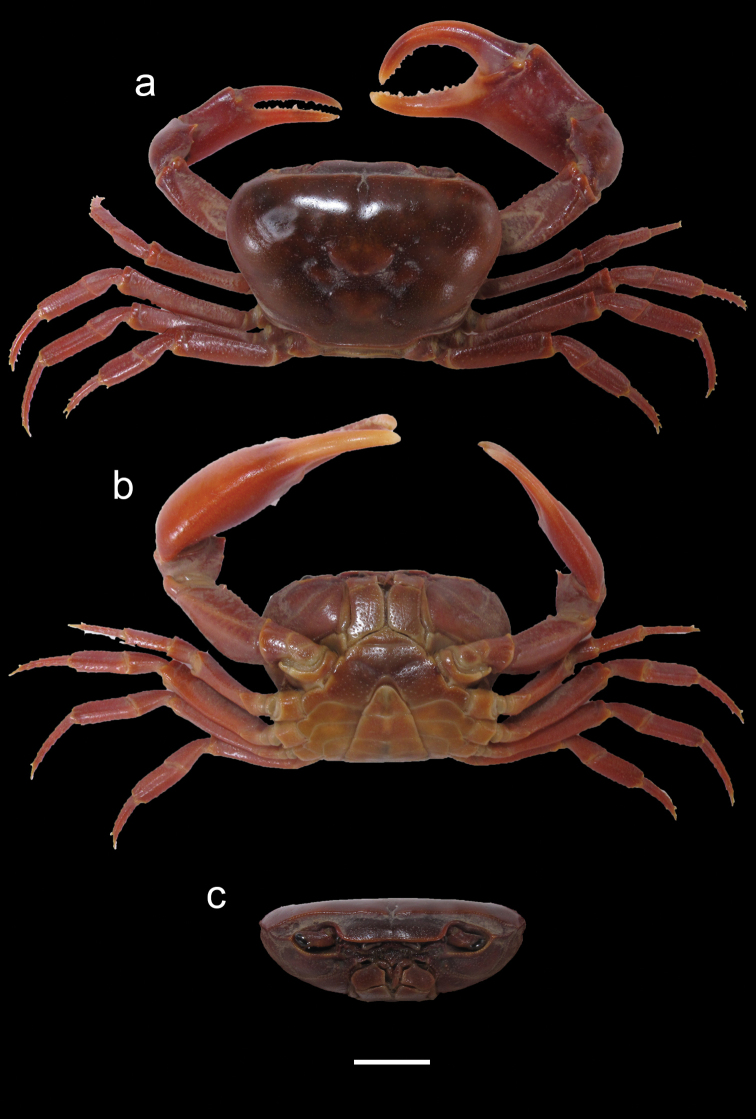
*Potamonautesamathole* sp. nov. male holotype (MB-A094813) **a** dorsal view **b** ventral view, and **c** cephalothorax, frontal aspect. Scale bar: 10 mm.

***Carapace*** (Fig. 4). Cephalothorax ovoid, dorsally flattened, maximum height and width at anterior third (CH/CL = 0.5, CWW/CL = 1.47). Branchial region rounded. Anterior margin straight, lying on same horizontal plane as anterolateral margin; anterolateral margin slightly granulated. Urogastric grooves well-defined; cardiac and cervical grooves well-defined where attached to urogastric groove, becoming poorly defined and shallow towards edge of carapace. Epigastric lobes well-defined by two indentations forked from midpoint of postfrontal crest. Postfrontal crest complete, straight, and distinct, curving down at epibranchial region, sloping backwards to join anterolateral margin. Exorbital teeth present. Epibranchial teeth absent. Carapace brown with orange-brown to purple-brown limbs when alive.

***Sternites*** (Fig. 4b). Sternites 1 and 2 fused, no sulcus. Second sulcus (s2/s3) prominent across sternum and third sulcus (s3/s4) complete, deep, projecting down medially towards abdomen.

**Figure 5.**
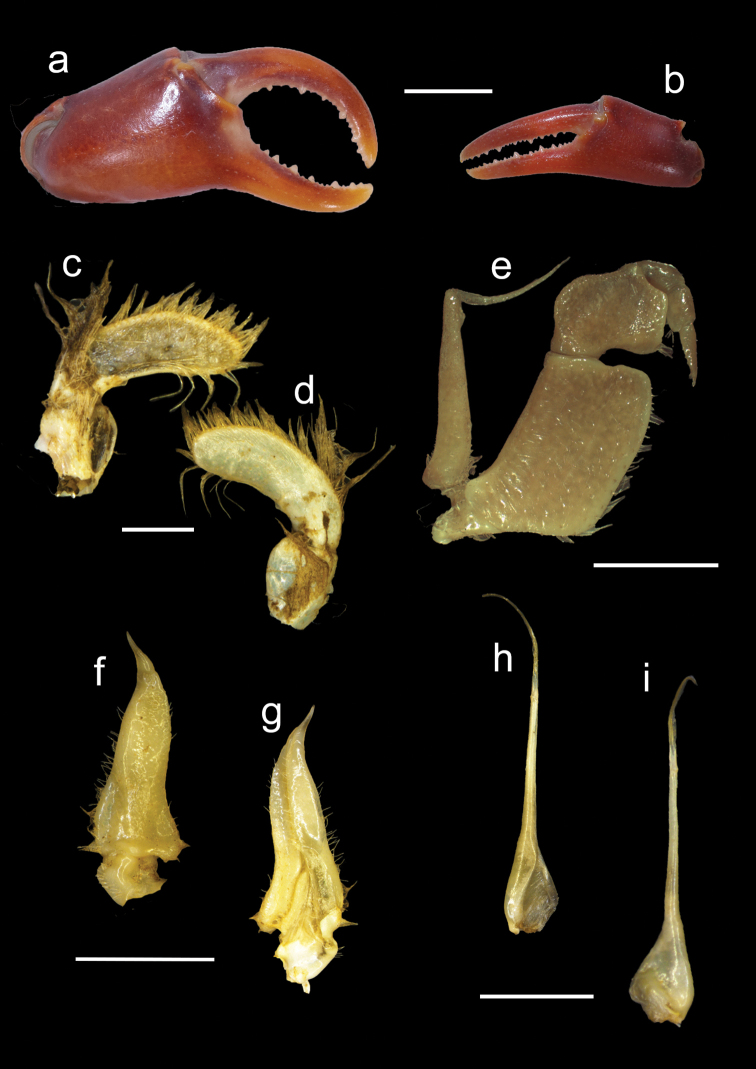
*Potamonautesamathole* sp. nov. male holotype (MB-A094813) **a** major cheliped **b** minor cheliped **c** right mandibular palp posterior view **d** right mandibular palp anterior view **e** 3^rd^ maxilliped **f** left gonopod 1 anterior view **g** left gonopod 1 posterior view **h** left gonopod 2 posterior view, and **i** left gonopod 2 anterior view. Scale bars: 10 mm (**a, b**); 1 mm (**c, d**); 5 mm (**e–i**).

***Third maxilliped*** (Figs 4c, 5e). Filling entire buccal frame except oval respiratory openings at top lateral corners. Ischium slightly scabrous, absence of vertical groove. Flagellum on exopod of third maxilliped curving upwards at distal ends.

***Mandibular palp*** (Fig. 5c, d). Consists of two segments. Terminal segment undivided, with dense tuft of setae on posterior proximal surface, margins hirsute. Subterminal segment short, thickened distally, almost round in appearance.

***Pereopods*** (Figs 4a, c, 5a, b). General right-handedness. Inequality of chelae (CRDL/CLDL = 1.41). Dactyl of major chela highly arched, large interspace formed in major cheliped when fingers closed, slim interspace formed when minor cheliped closed. Propodus fairly slim (CRPW/CRPL = 0.41), exhibiting 17 cutting teeth on major dactyl and 15 cutting teeth on pollex, some larger and more prominent. Carpi on either side with one prominent tooth and two rudimentary teeth. Meri granulated with spine on anterior surface. Slender pereopods (P2: ML/MW = 3.75; P5: ML/MW = 3.61), pereopod 4 longest, pereopod 5 shortest. Ventral margins of propodi smooth, dorsal margins bearing fine serration, dactyli serrated, ending in sharp points.

***Pleon*** (Fig. 4b). Somites 1–6 four sided, with triangular distally-rounded terminal segment (telson). First 5 somites broad and short; somite 6 and telson longer.

***Pleopods*** (Fig. 5f–i). Gonopod 1 terminal segment short, 0.24 length of subterminal segment, widest at base, tapering, ends in sharp point at distal end of terminal segment. Medial margin slightly irregular, inner lateral margin curved, margins hirsute. Terminal segment curves away from medial line when viewed posteriorly. Longitudinal groove extending the length of both subterminal and terminal segment, visible on dorsal surface. Gonopod 2 consisting of two segments; terminal segment 0.57 times length of subterminal segment, filamentous; subterminal segment widest at base, tapering gently inward 0.4 of length, forming narrow process supporting terminal segment. Gonopod 2 with straight subterminal segment, terminal segment curves inward toward medial line.

##### Variation.

The major cheliped is not always distinctly arched, especially in females and juveniles.

##### Live colouration.

Colouration varies between orange-brown to a darker purple-brown when alive. Tips of the dactyli may be paler in colour, displaying as orange or paler brown/purple.

##### Distribution.

Currently known only from the Katberg State Forest, the Hogsback State Forest, Madonna and Child Falls and the Hogsback Arboretum, all situated in the Amathole Mountain Range in the Eastern Cape province of South Africa.

##### Etymology.

The species is named after the Amathole Mountains, part of the Winterberg-Amathole mountain range complex, located on the Great Escarpment in the Eastern Cape. It is currently thought to be endemic to this region. The isiXhosa name ‘Amathole’ translates to ‘calves’ in English and refers to the mountain range, the forest, and the municipal district.

##### Remarks.

*Potamonautesamathole* sp. nov. is genetically and morphologically most similar to the Western Cape small-bodied montane freshwater crabs, i.e., *P.brincki* (Bott, 1960), *P.parvispina* Stewart, 1997, *P.parvicorpus* Daniels, Stewart & Burmeister, 2001, and *P.tuerkayi* Wood & Daniels, 2016. Morphologically, the species can be most easily distinguished from *P.parvispina* by the latter’s small but pronounced epibranchial tooth. *Potamonautesparvicorpus* bears slightly arched chelipeds, an arched carapace, and a poorly developed postfrontal crest, while the new species has highly arched major chelipeds, a flattened carapace and a distinct postfrontal crest. *Potamonautesbrincki* also has an arched carapace as well as a partitioned terminal segment of the mandibular palp with a setae-covered flange. *Potamonautesamathole* sp. nov. has a unilobed terminal segment of the mandibular palp with no flange. Of all the Western Cape montane freshwater crabs, *P.tuerkayi* is the most similar to *P.amathole* sp. nov. However, *P.tuerkayi* has a sharply tapering subterminal segment of gonopod 2, forming a rounded subterminal base, while in *P.amathole* sp. nov. this tapering is gradual, forming a sloping instead of a rounded base. Geographically, the above-mentioned species are all confined to the Cape Fold Mountain region, with *P.amathole* sp. nov. being the first described small-bodied montane freshwater crab from the Eastern Cape part of the Great Escarpment.

*Potamonautesdepressus* and *P.clarus* are two species of highland river crabs in the Drakensberg Mountain range. Although superficially similar to *P.amathole* sp. nov. in terms of a flattened carapace and slender limbs, morphological differences do exist in the structure of the mandibular palp and carapace depression. In *P.clarus*, a bright orange species, the mandibular palp has a flange on the terminal segment, while this is absent in *P.amathole*. *Potamonautesdepressus* has an extremely flattened carapace, with CH/CL ranging from 0.38–0.43. In *P.amathole* sp. nov., this depression of the carapace is less extreme with a ratio ranging from 0.43–0.51. Both of these species are confined to fast-flowing rivers in the Drakensberg highlands. In most other *Potamonautes* spp., pereopod 3 is the longest. However, in *P.amathole* sp. nov., pereopod 4 appears to be the longest.

##### Habitat and ecology.

Hogsback and Katberg are both situated in the Keiskamma River catchment. Both sites consist of Southern Mistbelt Forest (FoZ 3), known to be tall, multi-layered, species-rich forests dominated by *Afrocarpusfalcatus*, *Celtisafricana*, *Calodendrumcapense*, *Veprislanceolata*, and *Zanthoxylumdavyi* (Mucina and Rutherford 2006). Within the forest and grassland, patches of Eastern Temperate Freshwater Wetlands (AZf3) can be found (Mucina and Rutherford 2006).

The Hogsback (Madonna and Child) Waterfall site is situated inside the Hogsback State Forest. The habitat is represented by a tall, high-flowing stream with different biotopes and pools rich in macro-invertebrate diversity, i.e., Ephemeroptera, Coleoptera, Hemiptera, Diptera, Trichoptera, Odonata, and Plecoptera (Griffiths et al. 2015). The substrate is largely bedrock with sand and large stones. The site is characterised by a diversity of flora species, including *Canthiumciliatum*, *Protorhuslongifolia*, *Afrocarpusfalcatus*, and *Scolopiamundii* (Hawley et al. 2004; Mucina and Rutherford 2006).

The second site is situated inside the Hogsback Arboretum Park, downstream from the 39 Steps Waterfall. The habitat is represented by a small stream with pools and the substrate is largely sand with rocks. The edge of the stream is represented by marginal vegetation (i.e., *Pseudoschoenusinanis*) with tree canopy cover and burrows (Griffiths et al. 2015). The site is characterised by a diversity of planted flora species.

The Katberg site is situated in the montane Katberg State Forest. This habitat is comprised of a trickling stream on a very steep slope. The habitat is represented by low and clean water with some other macro-invertebrate diversity, i.e., Ephemeroptera, Coleoptera, Hemiptera, Diptera, Trichoptera, and Odonata (Griffiths et al. 2015). The substrate is largely bedrock with sand and stones. The edge of the stream is represented by marginal vegetation with dense tree canopy cover and burrows. The site is characterised by a diversity of flora species, including *C.ciliatum*, and *P.longifolia* (Hawley et al. 2004; Mucina and Rutherford 2006).

At the Hogsback Arboretum site, *P.amathole* sp. nov. co-occurs with *P.danielsi*. *Potamonautesdanielsi* is also found at the nearby Municipal Dam.

## ﻿Discussion

Resolving the distribution of southern African potamonautids is often difficult due to the general cryptic nature of this genus (Daniels et al. 2022). However, despite this, *Potamonautesamathole* sp. nov. can be distinguished from its sister species both genetically and morphologically. Providing detailed descriptions and highlighting morphological differences also facilitates accurate species records and highlights potentially new species, not just by the scientific community but also from citizen scientist observations (Daniels et al. 2022). Considering that more extensive sampling is required to fully explore potamonautid diversity, especially in high altitude and under-sampled regions, these citizen science observations play a significant role in the taxonomy of freshwater brachyurans.

16S Sequence divergences among previously known species ranged from 2.1 to 16.9%; the exception being the comparison between *P.dentatus* and *P.mhlophe* (1.5%). The indication of the *P.danielsi* Hogsback population, alongside the morphological identification of the specimen used in a study by Gouws et al. (2018), extends the known range of *P.danielsi* (see Peer et al. 2017) further south-westward and into the broader Amathole Forest and the Albany Thicket vegetation biomes (sensu Mucina and Rutherford 2006).

Although South African freshwater crabs have been extensively researched, we continue to find and describe new species (Daniels et al. 2021, 2022). This is partly due to ongoing efforts to target poorly sampled areas. Although the group has been well-studied in the KwaZulu-Natal and Western Cape provinces, this paper describes only the second montane freshwater crab from the Eastern Cape (the first being *P.baziya*). In KZN, two montane freshwater crabs, i.e., *P.clarus* and *P.depressus*, together with *P.baziya* represent the Drakensberg Escarpment species, with all three being closely related. In the Western Cape, *P.parvispina*, sister species to *P.amathole* sp. nov., is located within the Cederberg region. Myburgh and Daniels (2022) considered the speciation of the three additional Western Cape montane species, i.e., *P.brincki*, *P.tuerkayi*, and *P.parvicorpus*, and found an east/west separation defined by the Cape Fold Mountains. The results from this study and that of Daniels et al. (2021) indicate that *P.amathole* sp. nov. diverged before the separation of the Cape Fold montane species. Several routes of palaeo- and current connectivity along the Great Escarpment have been demonstrated for floristic species (Clark et al. 2011a, b; Janks 2014) with Galley et al. (2007) proposing a radiation from the Cape in a north-east direction towards the Drakensberg mountains, passing through the Winterberg-Amathole mountain range. A similar pattern of connectivity has been proposed for some invertebrate species including coleopterans (Bilton 2015) and ephemeropterans (Taylor et al. 2020), although this requires more in-depth investigation.

Barnes and Daniels (2019), in a study of onychophoran velvet worms from forests in the Winterberg-Amathole mountain range, highlighted that connectivity is often disrupted by forest fragmentation, especially in poorly conserved areas. This reinforces the need to target the under-sampled regions of South Africa, including the Great Escarpment, in order to properly describe biodiversity along this gradient and understand the phylogeographic patterns that occur along this system. This information, especially regarding endemic species, is crucial to highlight the need for more effective protection of South Africa’s freshwater systems.

## Supplementary Material

XML Treatment for Potamonautes amathole

## Supplementary materials

Supplementary material 1 Supplementary infromation Data type phylogenetic Explanation note **table S1**: Representatives of valid southern African *Potamonautes* species included in the phylogenetic analysis used to contextualise divergences and confirm the affinities of specimens included in the current study. Four species of *Maritomonautes* were included as outgroup taxa. GenBank accession numbers for the 16S rRNA and cytochrome c oxidase subunit I (COI) included and the data sources are provided; **table S2**: Uncorrected sequence divergences, expressed as percentages, as calculated from a 549 nucleotide alignment of 16S rRNA mtDNA sequences (below diagonal) and a 660 nucleotide alignment of the protein-coding mtDNA cytochrome c oxidase subunit I (COI) fragment (above diagonal), between representative of *Potamonautes* included in the present study. Newly-sampled specimens are identified as *Potamonautesdanielsi* and *P.amathole* sp. nov. https://binary.pensoft.net/file/846241This dataset is made available under the Open Database License (http://opendatacommons.org/licenses/odbl/1.0/). The Open Database License (ODbL) is a license agreement intended to allow users to freely share, modify, and use this Dataset while maintaining this same freedom for others, provided that the original source and author(s) are credited.Gavin Gouws
